# Cardiac tamponade caused by acute coxsackievirus infection related pericarditis complicated by aortic stenosis in a hemodialysis patient: a case report

**DOI:** 10.1186/s40792-018-0550-0

**Published:** 2018-12-06

**Authors:** Azumi Hamasaki, Tetsuro Uchida, Atsushi Yamashita, Kentaro Akabane, Mitsuaki Sadahiro

**Affiliations:** Second Department of Surgery, Yamagata University Faculty of Medicine, 2-2-2 Iida-Nishi, Yamagata, 990-9585 Japan

**Keywords:** Acute pericarditis, Aortic stenosis, Hemodialysis, Coxsackievirus, Cardiac tamponade

## Abstract

**Background:**

Pericardial effusion is observed in the majority of viral pericarditis cases; however, viral pericarditis accompanied by a large effusion resulting in cardiac tamponade is rare.

**Case presentation:**

Here, we report the case of a 75-year-old hemodialysis patient with acute viral pericarditis complicated by aortic stenosis. The patient was referred with a diagnosis of aortic stenosis and pericardial effusion. The pericardial effusion had increased during the preoperative examinations, and the inflammatory reaction had progressed. We decided to abort the surgical intervention and start oral administration of anti-inflammatory agents. We subsequently performed a pericardiocentesis. High antibody titers to coxsackievirus were noted in the pericardial effusion. Since no recurrence of the pericardial effusion was observed, the patient underwent an aortic valve replacement 2 months later. The pericardium completely adhered to the heart. Pathological examination of the pericardium showed fibrous pericarditis without active inflammation.

**Conclusions:**

Here, we successfully treated a hemodialysis case with severe aortic stenosis complicated by cardiac tamponade and worsened by acute viral pericarditis. We waited for the pericarditis to resolve after administering anti-inflammatory agents and performed pericardial drainage before carrying out aortic valve replacement. The perioperative course of our case was unique and suggestive.

## Background

Pericarditis can be a manifestation of infection, connective tissue disorders, malignancy, hypothyroidism, trauma, and end-stage renal dysfunction (ESRD) [1]. Pericarditis in ESRD patients can be categorized into uremic and dialysis-associated types [2]. Uremic pericarditis usually occurs before renal replacement therapy, whereas dialysis-associated pericarditis typically occurs in poorly controlled hemodialysis patients. On the other hand, pericardial effusion is observed in more than half of viral pericarditis cases; however, viral pericarditis accompanied by a large effusion resulting in cardiac tamponade is rare [3].

Here, we report the case of a 75-year-old hemodialysis patient with acute viral pericarditis complicated by aortic stenosis (AS). In this case, it was difficult to distinguish whether the pericarditis etiology was viral, hemodialysis-associated, or due to other causes. The perioperative course of our case was unique and suggestive.

## Case presentation

**Fig. 1:**
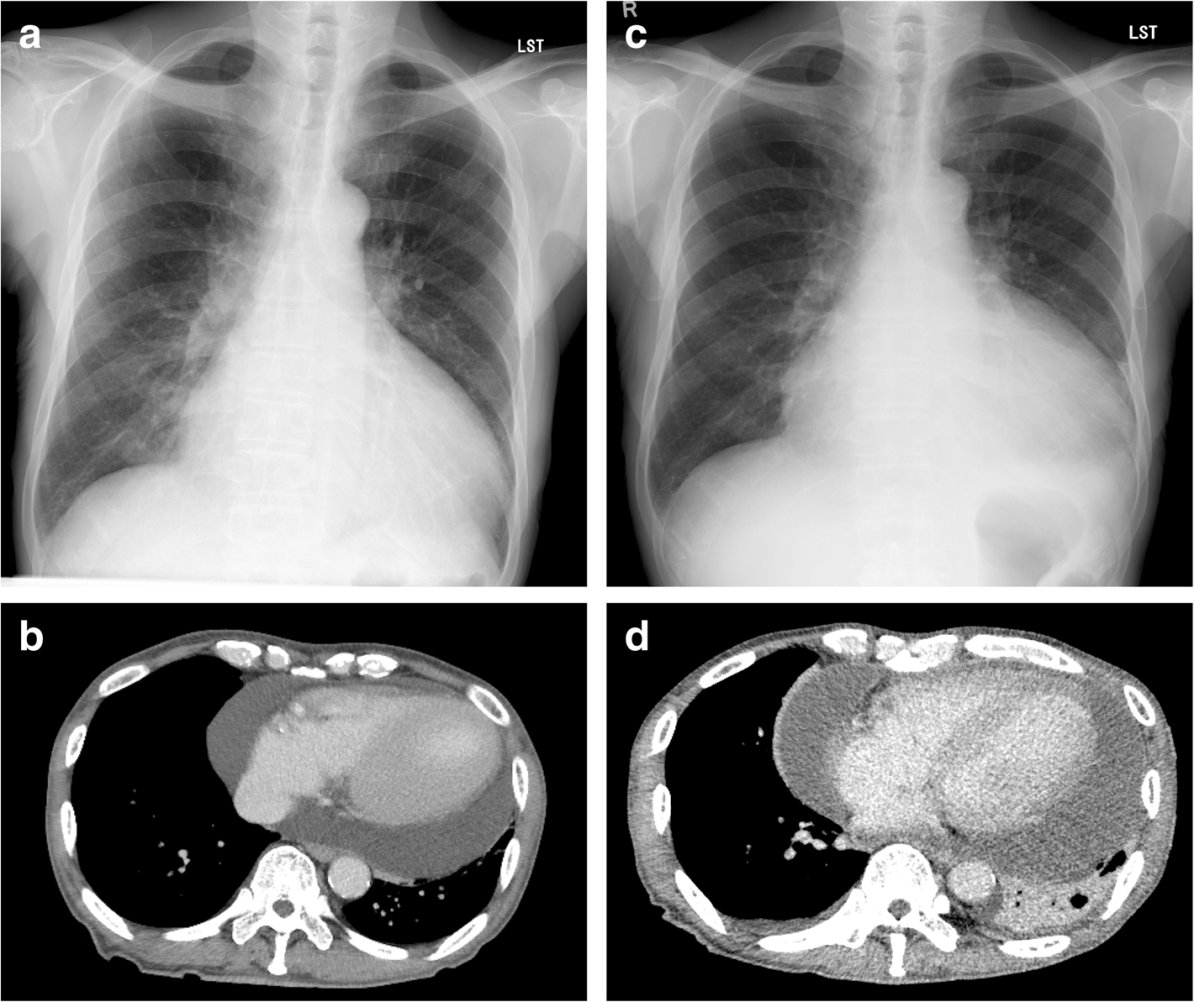
Severe cardiomegaly revealed on chest radiography at the initial outpatient appointment (**a**). Chest computed tomography showed a large pericardial effusion with an estimated volume of 700 mL (**b**). Cardiomegaly worsened 4 weeks after the initial appointment (**c**). Chest computed tomography showed a huge pericardial effusion with an estimated volume of 1300 mL (**d**)

A 75-year-old male taxi driver was referred with a diagnosis of severe AS and moderate pericardial effusion. He had a history of ESRD of unknown origin and had been on hemodialysis for 4 years. His primary complaint was dyspnea on effort that had gradually worsened in the previous 3 months. Electrocardiography revealed sinus rhythm, and chest radiography showed cardiomegaly with a cardiothoracic ratio of 68% (Fig. 1a). Transthoracic echocardiography (TTE) showed pericardial effusion and severe AS with peak and mean pressure gradients of 89 and 48 mmHg, respectively, and an aortic valvular area of 0.86 cm^2^. A medium volume of pericardial effusion was also observed on computed tomography (CT) (Fig. 1b). The estimated volume of the pericardial effusion was 700 mL. An aortic valve replacement (AVR) was scheduled. When he was admitted for the AVR 4 weeks after the initial outpatient counseling, he suffered from cough and fever. His white blood cell (WBC) count was 7700/μL and serum C-reactive protein (CRP) level was 9.0 mg/dL. Thus, the operation was postponed. When he was readmitted 2 weeks later, he had general fatigue and persistent inflammation was observed (WBC, 6250/μL; CRP, 6.84 mg/dL). Hepatic dysfunction (aspartate transaminase, 624 U/L; alanine transaminase, 1072 U/L) was newly detected. Electrocardiography revealed paroxysmal atrial fibrillation. The cardiothoracic ratio increased to 71% on chest radiography (Fig. 1c). Massive pericardial effusion was observed on chest CT (Fig. 1d). The estimated pericardial effusion volume was 1300 mL. A diagnosis of cardiac tamponade was made. We decided to abort the operation and started the administration of oral loxoprofen at 300 mg/day. Pericardiocentesis was performed, and a total of 1084 mL of bloody pericardial effusion was successfully drained. Examination of the fluid revealed antibody titers to coxsackievirus A4 and B4 elevated 256 times above the normal range. Several tumor marker levels in the pericardial effusion were also elevated (carcinoma antigen-125, 384.9 U/mL; cytokeratin 19 fragment, 43.5 ng/mL); however, cytology of the fluid revealed no malignancy. No abnormal accumulation of fluorodeoxyglucose was detected on positron emission tomography–CT. The inflammation improved immediately. During a 2-month outpatient observation, the inflammation was well-controlled and no recurrence of the pericardial effusion was observed. Thus, the AVR was rescheduled.

The elective operation was performed. The pericardium was thickened and showed almost complete adherence to the heart. Following meticulous dissection of the adhesions, isolation of the right pulmonary veins was performed with a radiofrequency ablator (Isolator Synergy Clamp OSL2; AtriCure Inc., West Chester, OH, USA) before the initiation of cardiopulmonary bypass (CPB). After the establishment of CPB, dissection of the adhesion around the left pulmonary veins was performed and the left pulmonary veins were similarly isolated. Myocardial protection was achieved using intermittent antegrade and retrograde cold blood cardioplegia. The aortic valve was exposed via an aortotomy. A visual inspection revealed calcified cusps and annulus. The cusps were resected, and the calcifications on the annulus were gently removed using an ultrasonic aspirator (Sonopet UST-2001; Stryker Japan Inc., Tokyo, Japan). A 23-mm stentless bioprosthesis (Solo Smart; Sorin Group, Milan, Italy) was implanted with three continuously running 4–0 monofilament polypropylene sutures. Aortic cross-clamp time and CPB time were 72 and 127 min, respectively. The patient tolerated the procedure well and had an uncomplicated postoperative course. Postoperative TTE showed no aortic regurgitation. The effective orifice area (EOA) and indexed EOA of the bioprosthesis were calculated as 1.73 cm^2^ and 1.08 cm^2^/m^2^, respectively, and mean pressure gradient across the prosthetic valve was 10 mmHg. Anti-platelet therapy (aspirin 100 mg/day) was initiated on the first postoperative day. The patients had no adverse events during 1 year and 9 months of follow-up, and his sinus rhythm has been maintained.

**Fig. 2:**
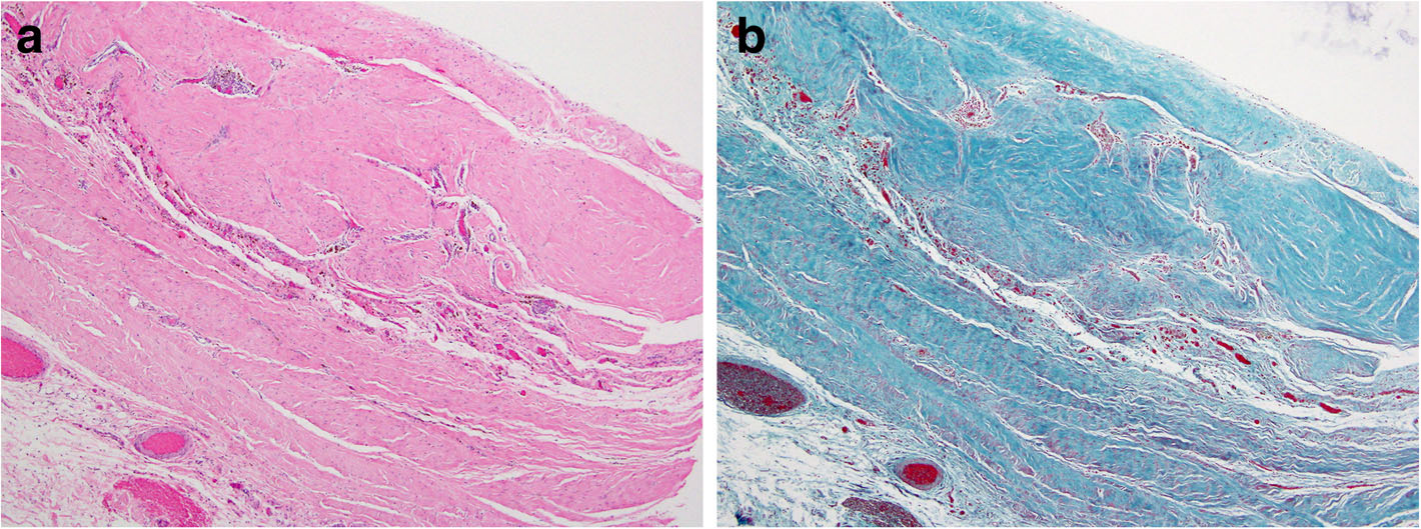
Pathological examination revealed fibrosis and scar formation in the thickened pericardium. Mild perivascular infiltration of T-lymphocytes was observed. Collagen fiber complex formation was increased as demonstrated by a finding of hyalinization. Edematous changes or fibrin precipitation were not observed. **a** Hematoxylin-eosin staining. **b** Elastica-Masson staining

Pathological examination of the pericardium (Fig. 2) showed fibrous pericarditis without active inflammation. There was no malignancy in the immune-stained specimen. We could not detect the specific cause of the pericarditis in the pathological specimen.

## Discussion

Viral pericarditis accounts for the majority of acute pericarditis cases and is rarely life-threatening. Pericarditis can result from the direct viral infection of the pericardium, most commonly adenovirus or coxsackievirus [3, 4]. Bacterial pericarditis is also recognized as an infectious pericarditis that usually results from direct or hematogenous spread from other sites of infection, such as pneumonia, osteomyelitis, or meningitis. Tuberculous pericarditis is a special type of bacterial pericarditis that is strongly associated with the development of constrictive pericarditis. Pericarditis can also be a manifestation of connective tissue disorders, malignancy, hypothyroidism, trauma, and ESRD [1].

Pericarditis in ESRD patients can be categorized into uremic and dialysis-associated types [2]. Uremic pericarditis is characteristically described as the onset of pericarditis before renal replacement therapy or within 8 weeks of its initiation, whereas dialysis-associated pericarditis typically occurs after 8 weeks of dialysis. The reported incidence of dialysis-associated pericarditis is 2–21%. Uremic pericarditis usually resolves with dialysis and rarely causes complications. Dialysis-associated pericarditis is rare in well-controlled hemodialysis patients. Inadequate dialysis and/or fluid overload may contribute to this pathology. Banerjee and Davenport suggested that when ESRD patients on adequate dialysis develop pericarditis, early pericardial drainage rather than intensifying dialysis should be considered [5]. Since our patient had been on well-controlled dialysis, we considered the possibility of dialysis-associated pericarditis to be low.

Pericardial effusion is observed in 60% of viral pericarditis cases; however, viral pericarditis accompanied by a large effusion resulting in cardiac tamponade is rare [3]. There have been only four reports of cardiac tamponade due to acute pericarditis with coxsackievirus [6–9]. Although our patient had elevated antibody titers to coxsackievirus as well as elevated levels of several tumor markers in the pericardial effusion, we could not completely rule out pericarditis caused by dialysis or a malignant neoplasm. It was difficult to identify the cause of the pericarditis; thus, we hesitated to perform early AVR at the initial admission.

Based on the above information, the large pericardial effusion that caused the cardiac tamponade in this case was considered to be due to a combination of multiple causes such as severe AS, chronic heart failure, and repeated volume overload due to intermittent hemodialysis, which had caused a medium pericardial effusion. The pericardial effusion increased rapidly after the onset of viral pericarditis, resulting in progression of heart failure and cardiac tamponade.

During the observation, heart failure improved with a single pericardial drainage. The inflammatory response resolved, and recurrence of the pericardial effusion was controlled by anti-inflammatory drugs. On the other hand, the pericardium was tightly adhered to the heart, making the surgery difficult. In such complicated cases, it might be better to consider early surgery; however, it was difficult to decide the optimal surgical timing.

## Conclusion

Here, we successfully treated a hemodialysis patient with severe AS complicated by cardiac tamponade and worsened by acute viral pericarditis. We waited for the pericarditis to improve after administering anti-inflammatory agents and performing pericardial drainage and then performed the AVR. The perioperative course of our case was unique and suggestive.

## Abbreviations

AS: Aortic stenosis
AVR: Aortic valve replacement
CPB: Cardiopulmonary bypass
CRP: C-reactive protein
CT: Computed tomography
EOA: Effective orifice area
ESRD: End-stage renal dysfunction
TTE: Transthoracic echocardiography
WBC: White blood cell
